# Supplementary material for: Inhibition of glutaminase elicits senolysis in therapy-induced senescent melanoma cells
Source: Cell Death Dis. 2024 Dec 18;15(12):902. doi: 10.1038/s41419-024-07284-3 (PMC11655860; doi:10.1038/s41419-024-07284-3)
Supplement: Supplementary file 1 — Supplemental figures 1-10 [file 41419_2024_7284_MOESM1_ESM.pdf]

Supplemental Fig 1.

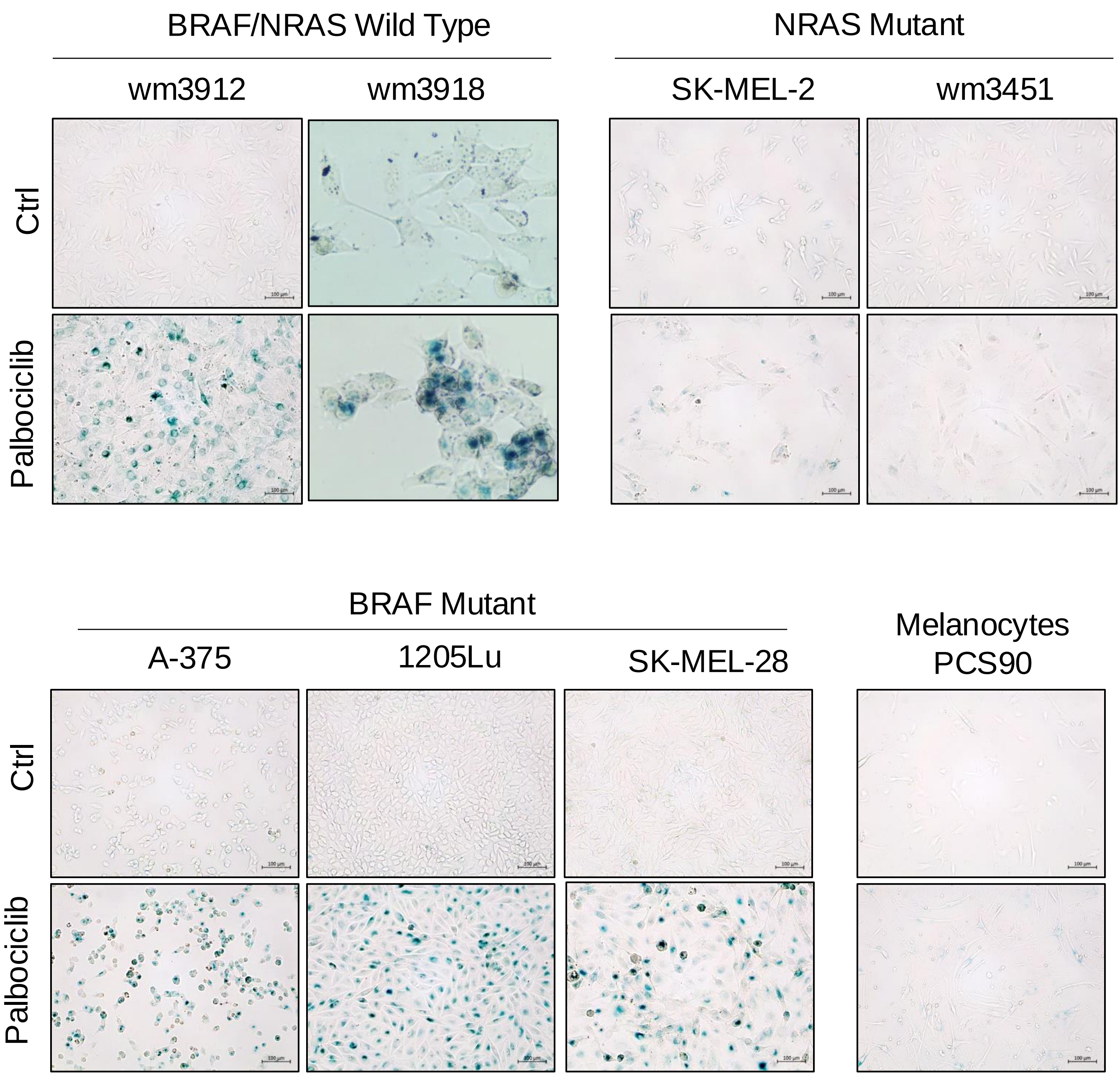

Fig S1. Palbociclib induces senescence in Nras WT melanoa.

WM3912 (Braf^{WT}Nras^{WT}), WM3918 (Braf^{WT}Nras^{WT}), A-375 (Braf^{V600E}Nras^{WT}), 1205Lu (Braf^{V600E}Nras^{WT}), Skmel28 (Braf^{V600E}Nras^{WT}), Skmel2 (Braf^{WT}Nras^{Q61R}), WM3451 (Braf^{WT}Nras^{Q61K}), and PCS90 (melanocytes) were treated with DMSO (Control) or palbociclib (1μM) for 8 days and subjected to SA-βgal staining. Representative images of SA-βgal staining are shown.

Supplemental Fig 2.

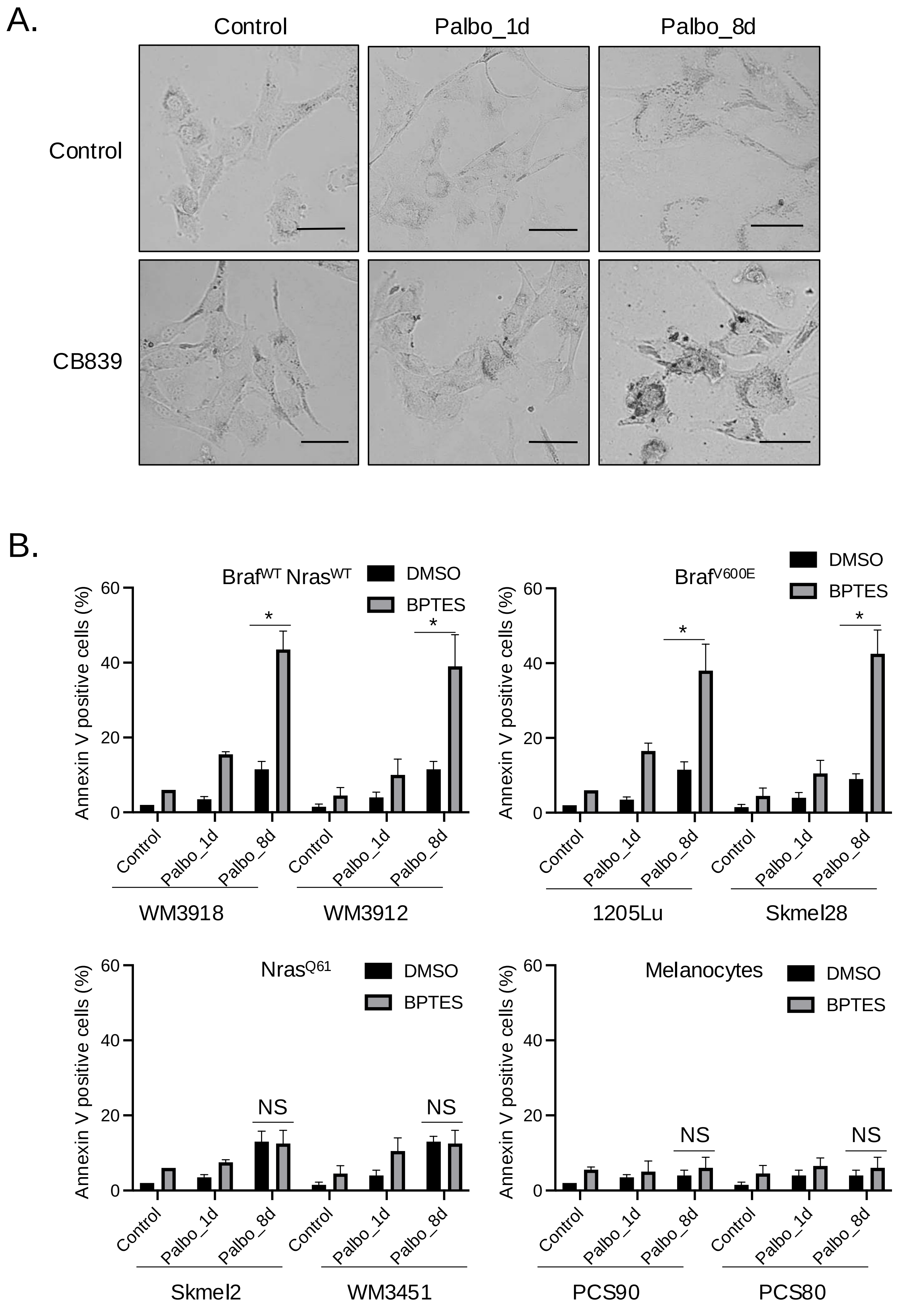

Fig S2. GLS1i induces senolysis in palbociclib-induced senescent cells.

(A) Skmel28 cells were treated with DMSO (Control) or palbociclib (1μM) for 1 day or 8 days followed by CB-839 (50μM) for an additional 3 days. Representative phase contrast images of each treatment were shown. Scale bar indicates 50μm. (B) WM3918 ($Braf^{WT}Nras^{WT}$), WM3912 ($Braf^{WT}Nras^{WT}$), 1205Lu ($Braf^{V600E}Nras^{WT}$), Skmel28 ($Braf^{V600E}Nras^{WT}$), Skmel2 ($Braf^{WT}Nras^{Q61R}$), WM3451 ($Braf^{WT}Nras^{Q61K}$), PCS90 (melanocytes) and PCS80 (melanocytes) were treated with DMSO (Control) or palbociclib (1μM) for 1 day or 8 days followed by BPTES (5μM) for an additional 3 days. Harvested cells were subjected to Annexin V staining and analyzed by flow cytometry. Data represent mean ± SD, * $p < 0.05$, NS non-significant.

Supplemental Fig 3.

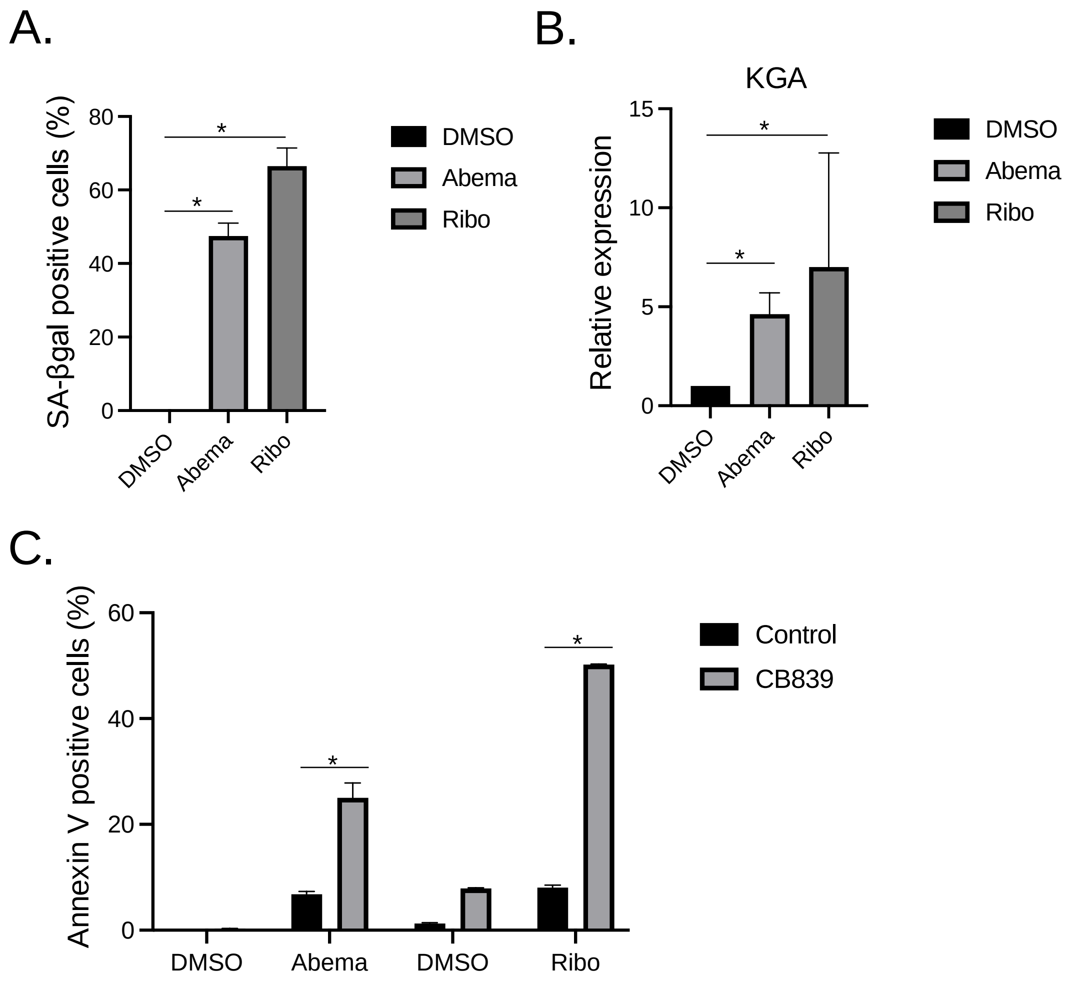

Fig S3. GLS1i induces senolysis in CDK4/6i-induced senescent cells.
(A-B) 1205Lu cells were treated with DMSO (Control), abemaciclib (Abema) (1μM) or ribociclib (Ribo) (1μM) for 8 days. Harvested cells were subjected to SA-βgal assay (A), RT-qPCR analysis for KGA (B). Data were normalized by GAPDH and represent mean ± SD, * $p < 0.05$ (two-tailed Student's t-test; n = 3). (C) 1205Lu cells were treated with DMSO (Control), abemaciclib (1μM) or ribociclib (1μM) for 8 days followed by CB-839 (50μM) for an additional 3 days. Harvested cells were subjected to Annexin V staining and analyzed by flow cytometry. Data represent mean ± SD, * $p < 0.05$.

Supplemental Fig 4.

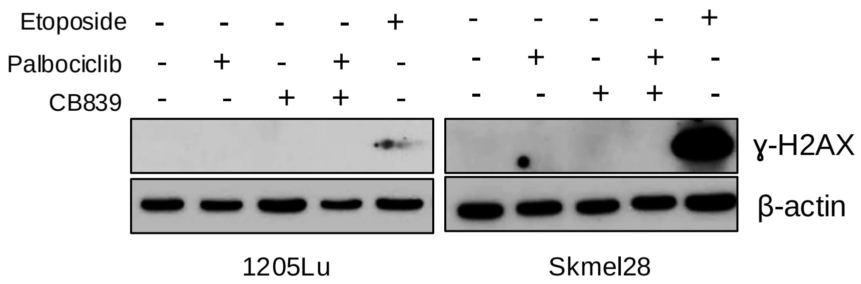

Fig S4. Palbociclib and CB-839 do not induce DNA damage.

(A) 1205Lu and Skmel28 cells were treated with DMSO (Control), palbociclib for 11 days, CB-839 for 3 days, or palbociclib (1μM) for 8 days followed by CB-839 (50 μM) for an additional 3 days. Etoposide treatment (10 μM) was used as a positive control. Harvested cells were subjected to western blot for ɣ-H2AX and β-actin.

Supplemental Fig 5.

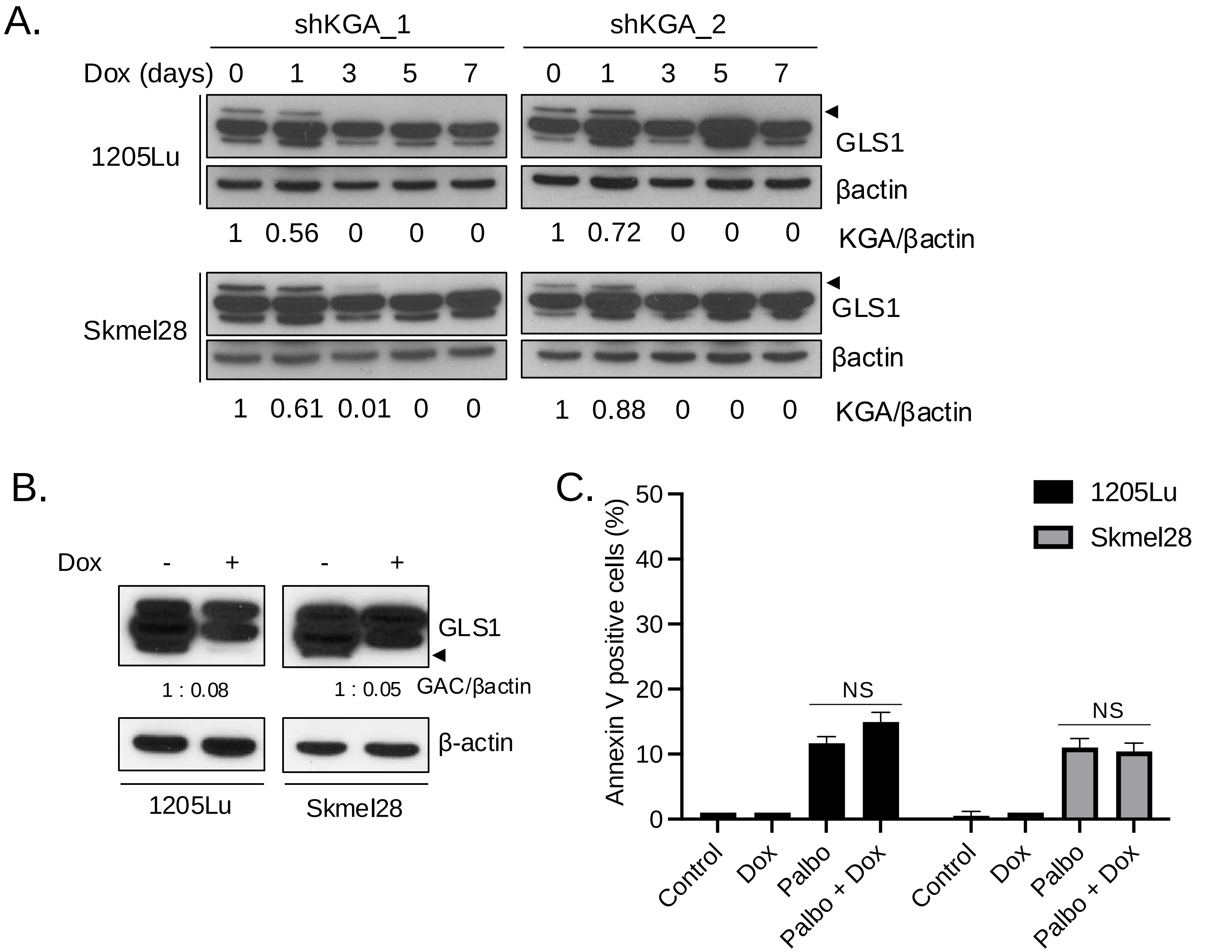

Fig S5. Manipulation of KGA and GAC expression in 1205Lu and skmel28 cells. (A) 1205Lu and Skmel28 cells were introduced with doxycycline-inducible shKGA (shKGA-1 or shKGA-2). After selection, cells were treated with doxycycline (100 ng/ml) for 1, 3, 5, and 7 days. Harvested cells were subjected to western blot for GLS1 and β-actin. Arrowheads indicate KGA expression. (B) 1205Lu and Skmel28 cells were introduced with doxycycline-inducible shGAC. After selection, cells were treated with doxycycline (100 ng/ml) for 5 days. Harvested cells were subjected to western blot for GLS1 and β-actin. Arrowheads indicate GAC expression. (C) Cells from (B) were treated with palbociclib (1μM) for 13 days, doxycycline (100 ng/ml) for 5 days, or palbociclib (1μM) for 8 days followed by doxycycline (100 ng/ml) for an additional 5 days. Harvested cells were subjected to Annexin V staining and analyzed by flow cytometry. Data represent mean ± SD, NS non-significant.

Supplemental Fig 6.

A.

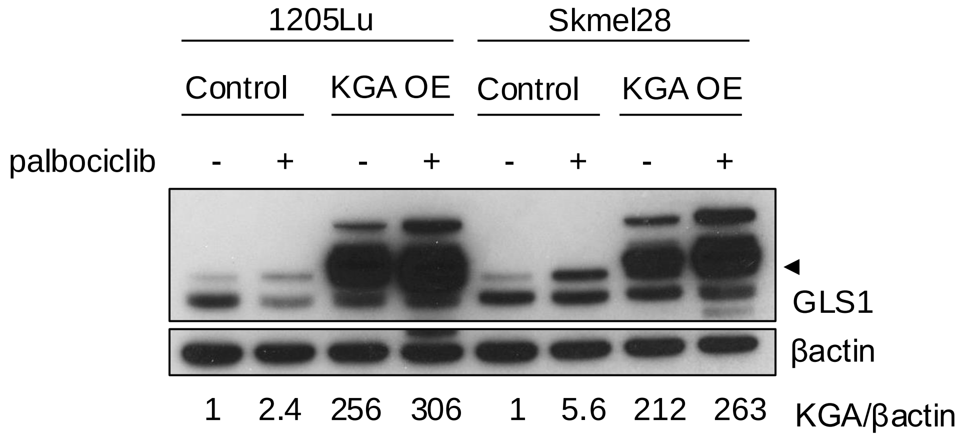

Fig S6. KGA upregulation after palbociclib treatment in 1205Lu and skmel28 cells. 1205Lu and Skmel28 cells introduced with or without KGA overexpression were treated with DMSO or palbociclib for 8 days. Harvested cells were subjected to western blot for GLS1 and β-actin. Arrowheads indicate KGA expression.

Supplemental Fig 7.

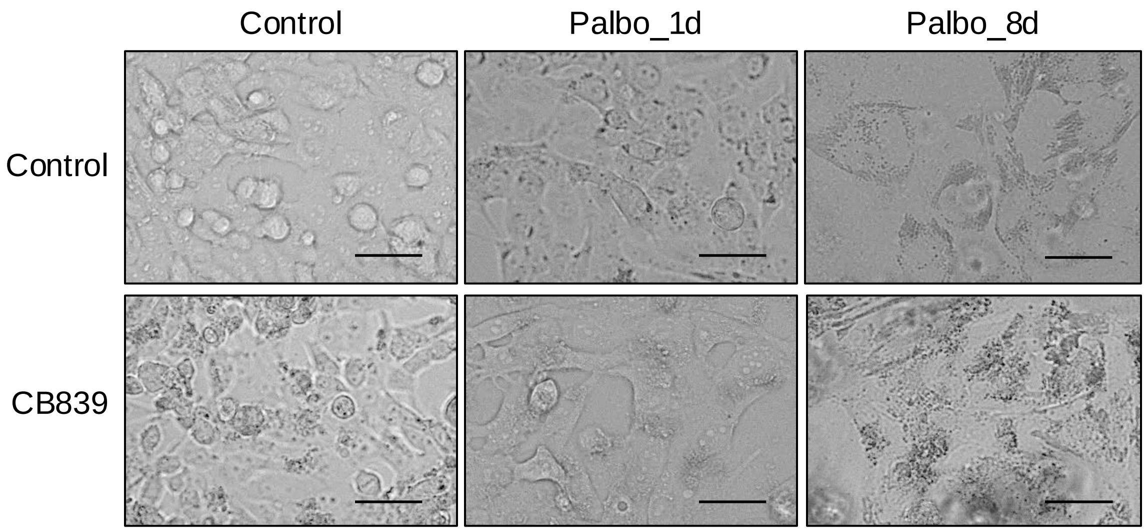

Fig S7. GLS1i induces senolysis in palbociclib-induced vemurafenib-resistant senescent cells.

1205Lu vemurafenib-resistant (VR) cells were treated with DMSO (Control) or palbociclib (1μM) for 1 day or 8 days followed by CB-839 (50μM) for an additional 3 days. Representative phase contrast images of each treatment are shown. Scale bar indicates 50μm.

Supplemental Fig 8.

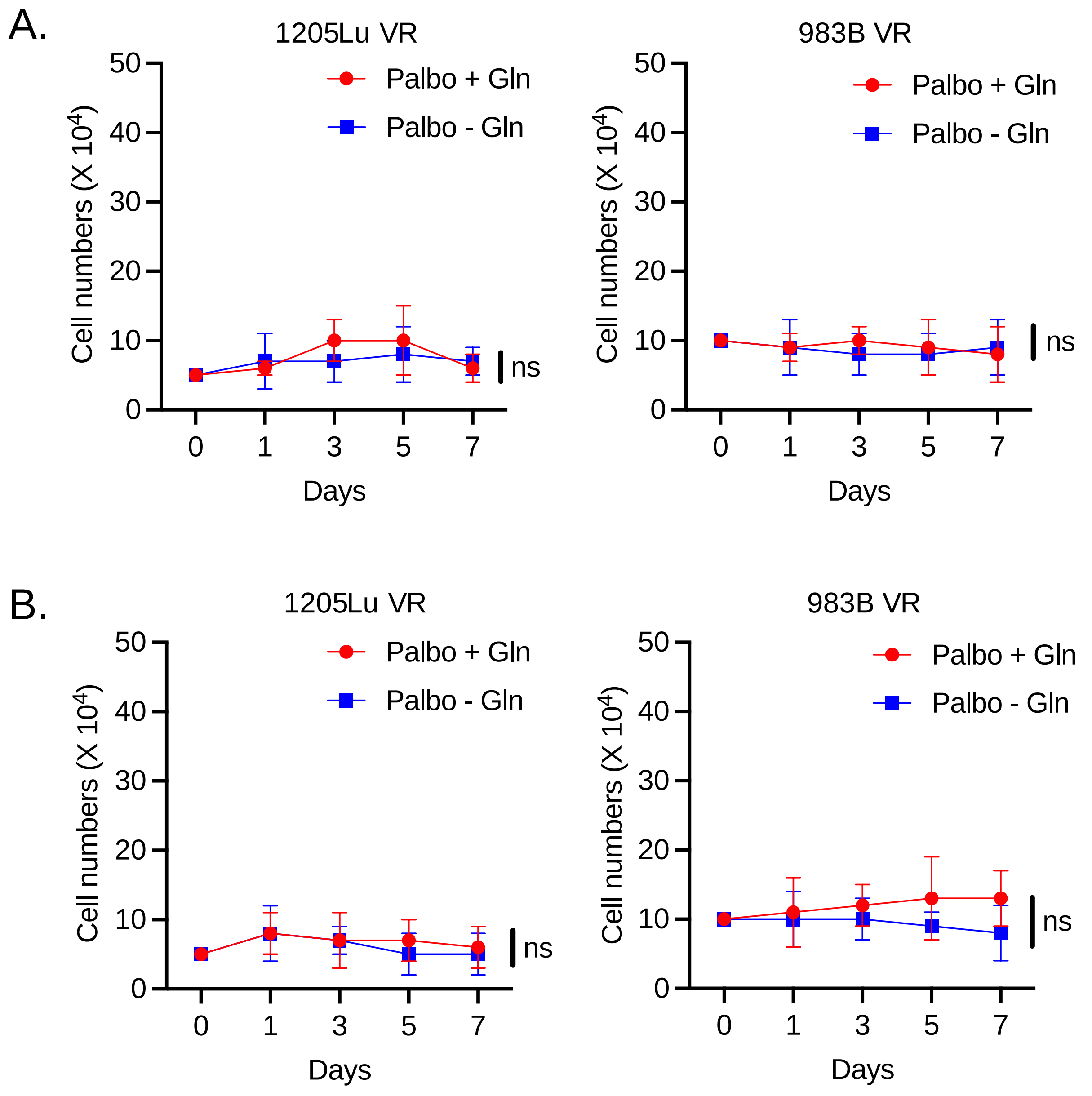

Fig S8. Palbociclib and Glutamine depletion do not induce senolysis.

(A) 1205Lu_VR or 983B_VR cells were treated with palbociclib (1μM) for 8 days and then cultured in glutamine-depleted medium. Cell numbers were counted at 1, 3, 5, and 7 days after glutamine withdrawal. (B) 1205Lu_VR or 983B_VR cells were simultaneously treated with palbociclib (1μM) in glutamine-depleted medium. Cell numbers were counted at 1, 3, 5, and 7 days after palbociclib treatment and glutamine withdrawal. Data represent mean ± SD, ns indicates non-significant (n = 3).

Supplemental Fig 9.

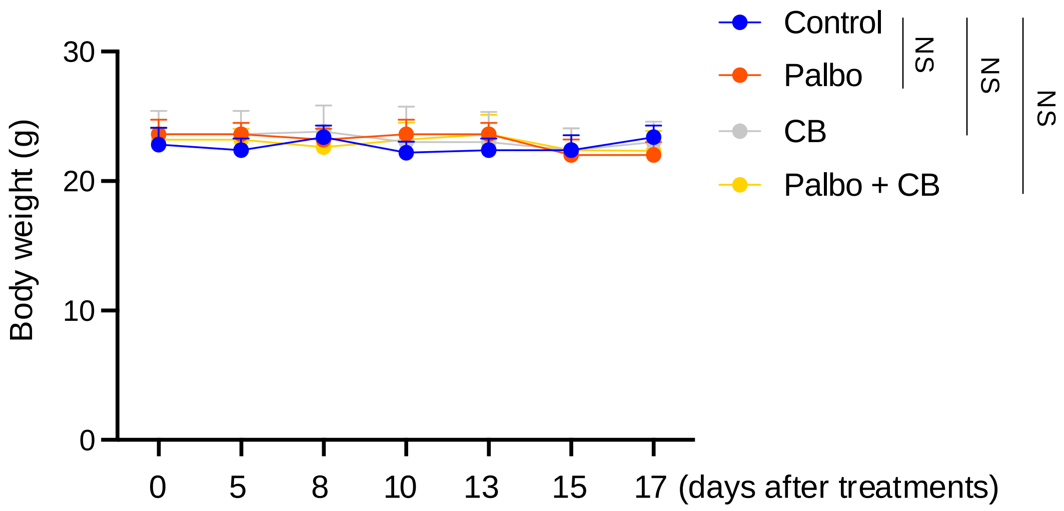

Fig S9. mouse body weights are unchanged after treatments.

Mouse body weights were measured at indicated time points in each mouse after treatments. NS indicates non-significant (n = 5).

Supplemental Fig 10.

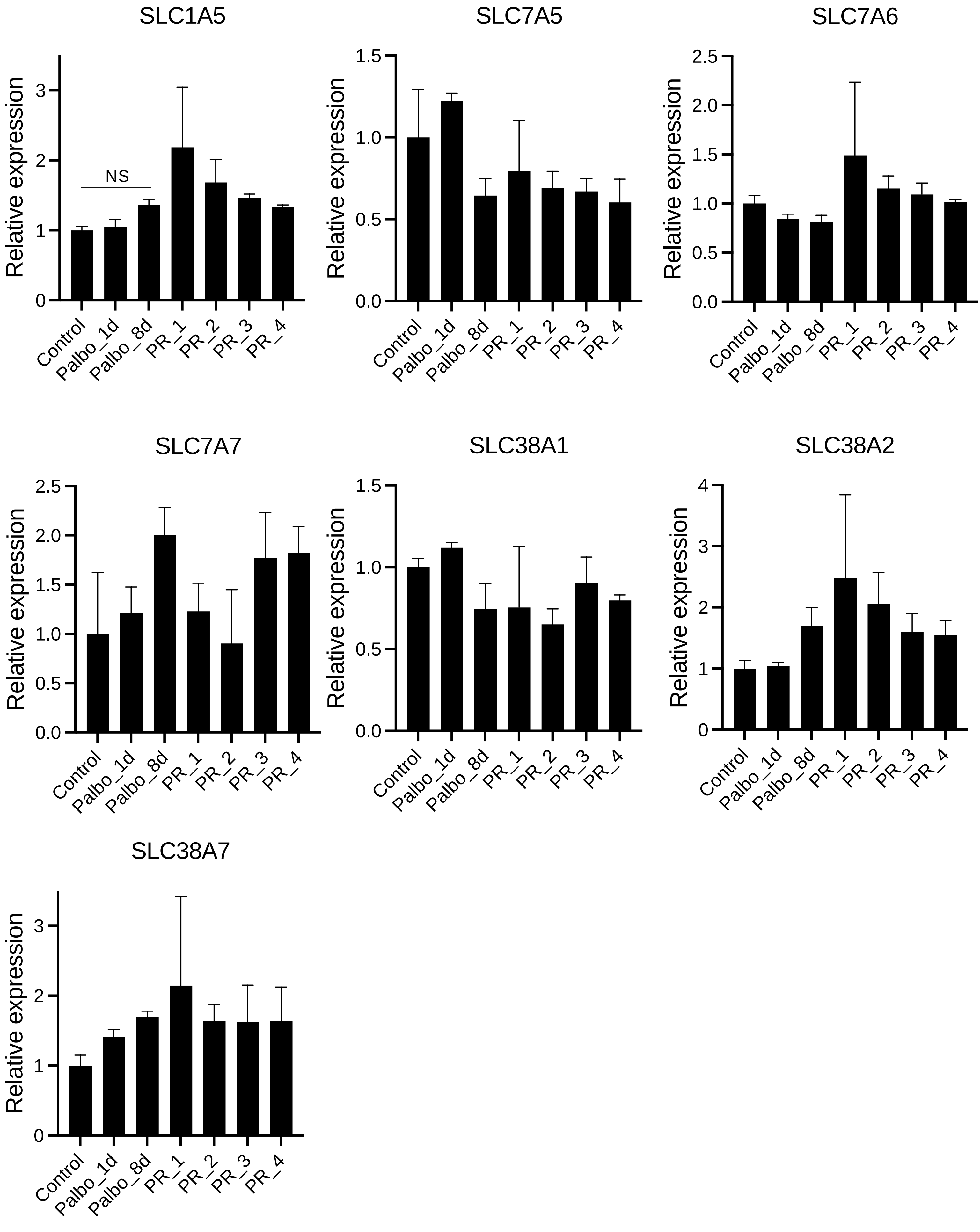

Fig S10. Expression of glutamine transport is unchanged.

(A) 1205Lu cells treated with DMSO (Control) or palbociclib (1μM) for 1 day or 8 days and 4 clones of 1205Lu palbociclib resistant cells (PR_1, PR_2, PR_3 and PR_4) were harvested and subjected to RNA-seq in a previous study (Yoshida et al, *Sci Adv* 2019). Data mining of RNA seq data for the expression of glutamine transporters is shown. Data represent mean ± SD.
