# Supplementary material for: Inhibition of glutaminase elicits senolysis in therapy-induced senescent melanoma cells
Source: Cell Death Dis. 2024 Dec 18;15(12):902. doi: 10.1038/s41419-024-07284-3 (PMC11655860; doi:10.1038/s41419-024-07284-3)

Manuscript WB Scans

Fig 1E

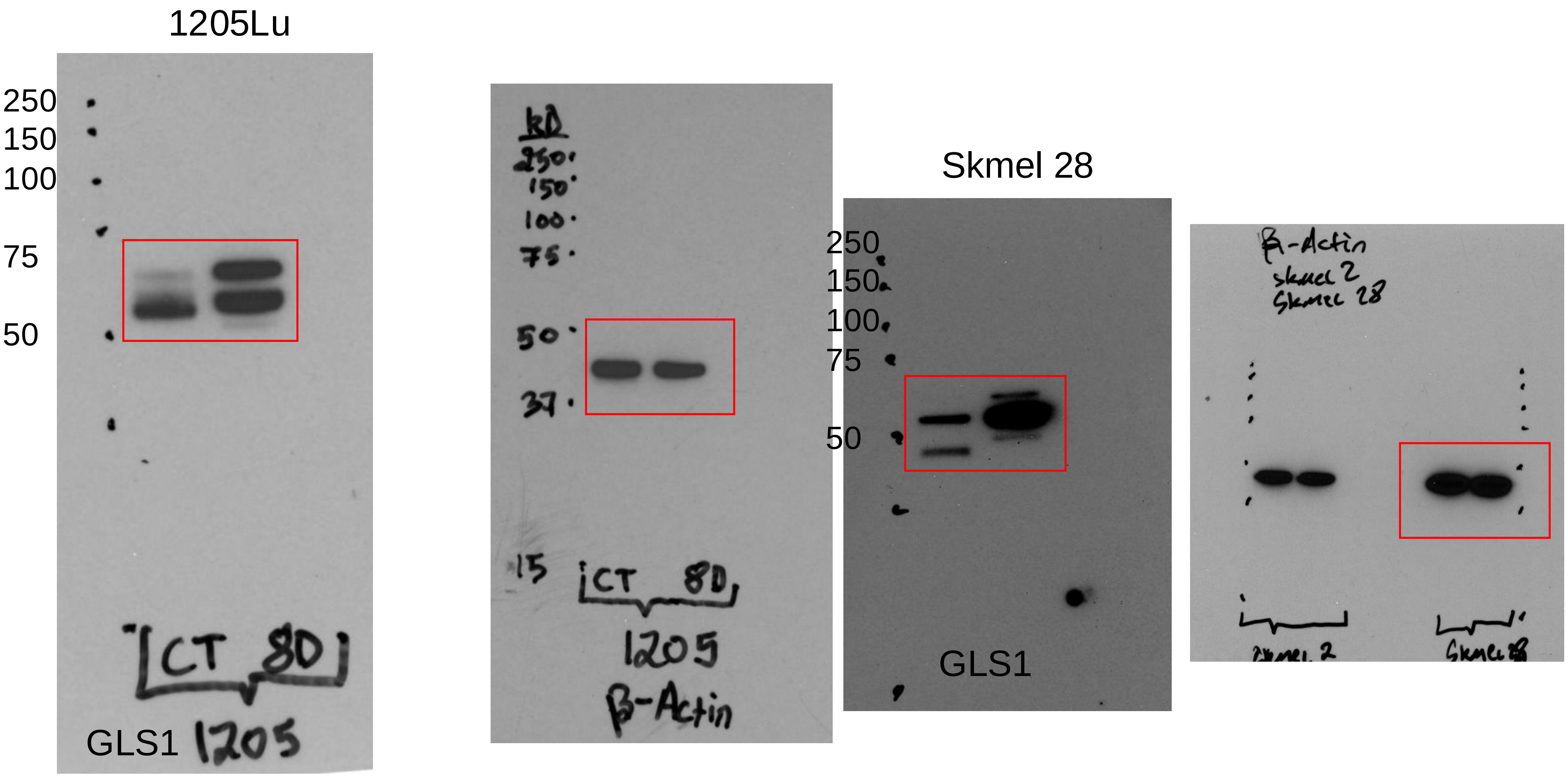

1205Lu
250
150
100
75
50
CT 8D
GLS1 1205
kD
250
150
100
75
50
37
15
CT 8D
1205
β-Actin
Skmel 28
250
150
100
75
50
GLS1
β-Actin
Skmel 2
Skmel 28

Fig. 3-A (Inducible shKGA in 1205, Skmel28)

File Name: “1205,Skmel28_shKGA_Beta Actin”

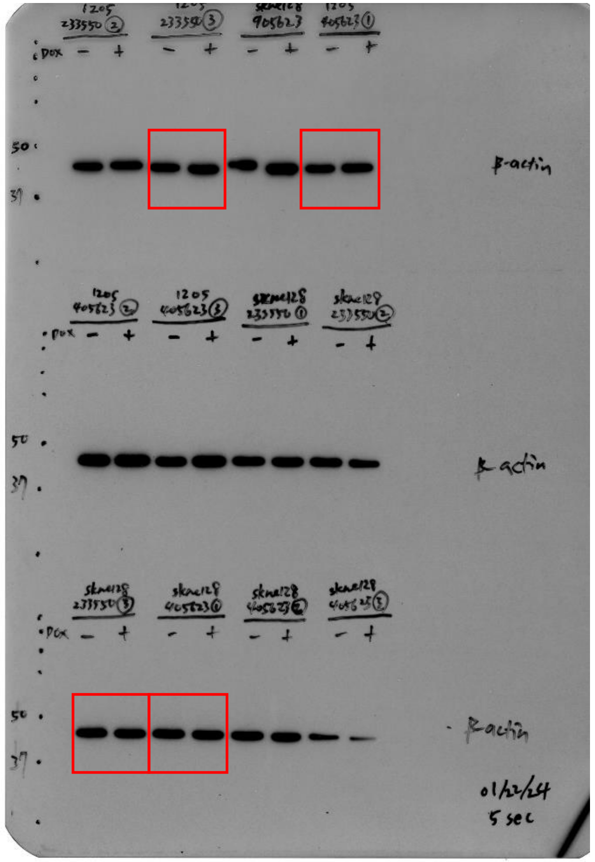

“1205_shKGA_1_GLS1”

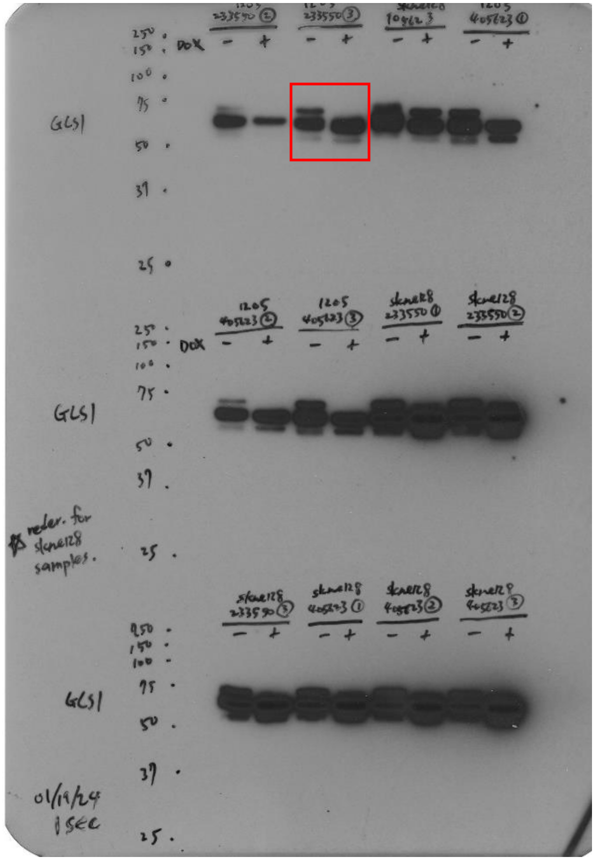

“1205_shKGA_2_Skmel28_shKGA_1,2_GLS1”

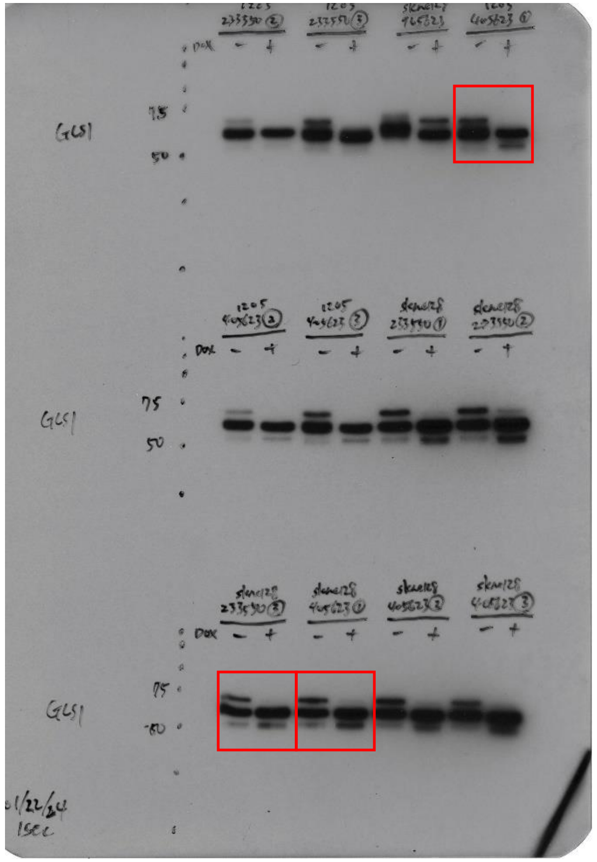

Fig. 3-C (KGAOE in 1205, Skmel28)

File Name:

"1205,Skmel28_KGAOE_Beta Actin"

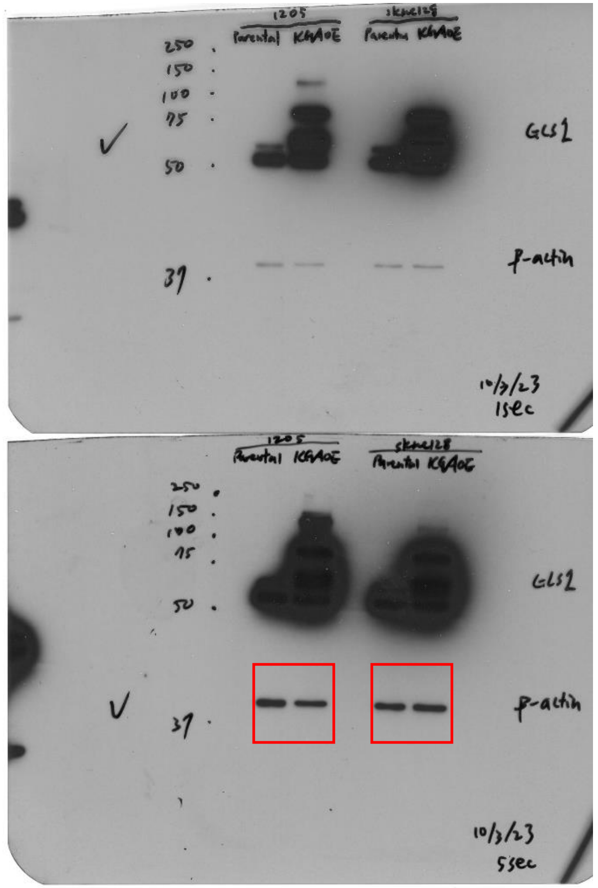

"1205,Skmel28_KGAOE_GLS1"

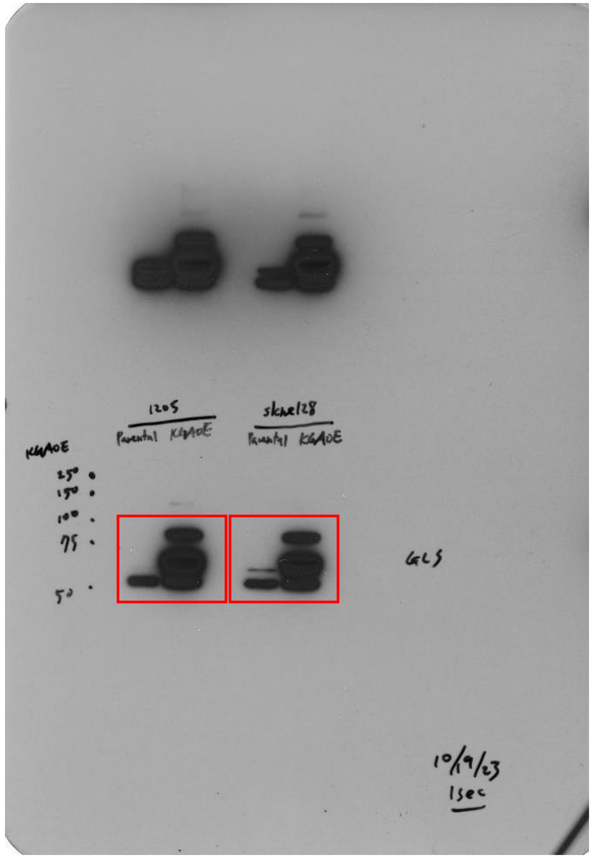

Fig 4B

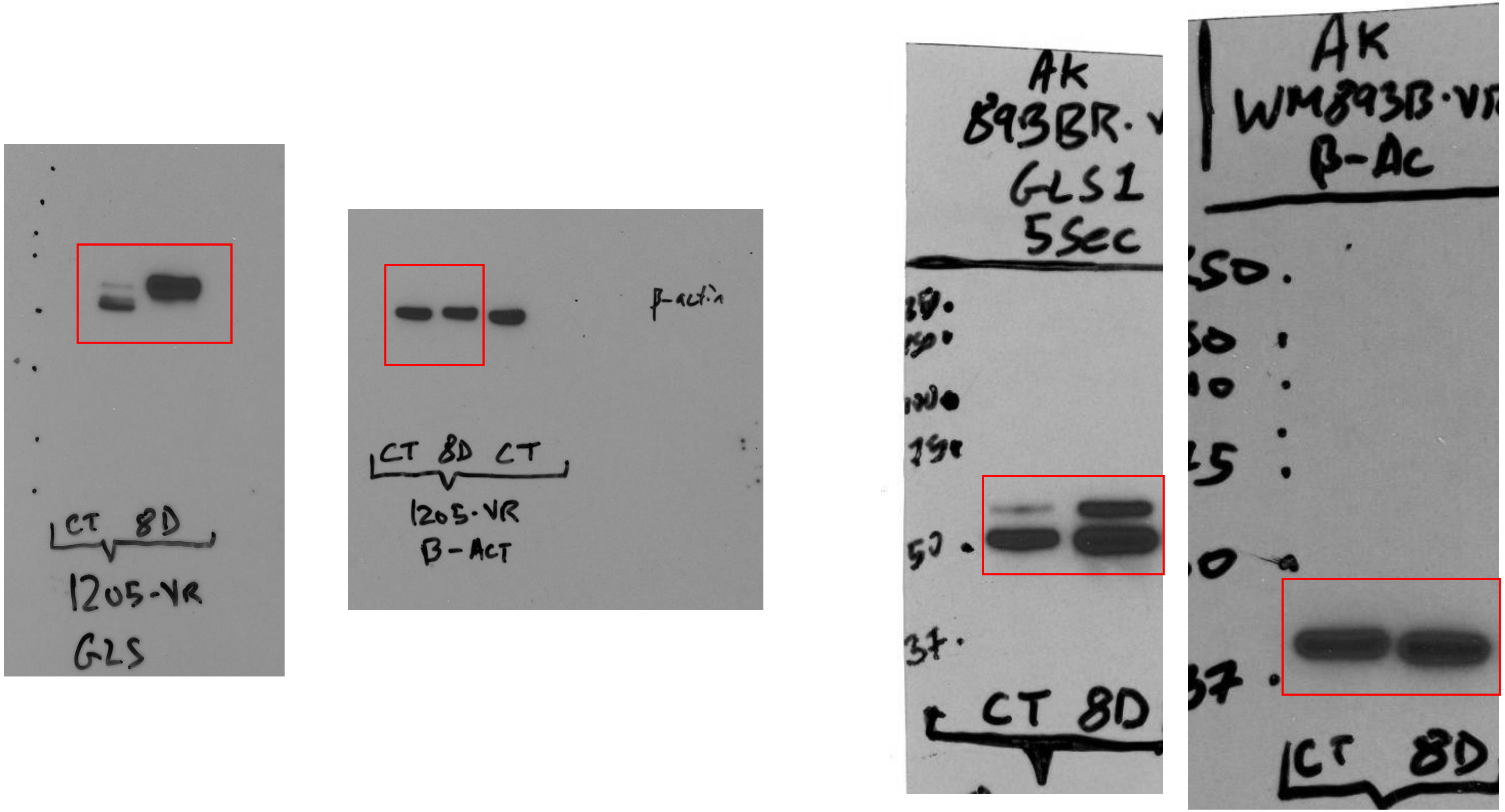
CT 8D
1205-VR
GLS
CT 8D CT
1205-VR
B-ACT
β-actin
AK
GLS1
5 Sec
CT 8D
AK
β-Ac
CT 8D

Fig. 6-C (10uM Vem 1D in 1205)

File Name: “1205_10uM Vem 1D _Beta Actin” “1205_10uM Vem 1D_P-ERK, P-S6” “1205_10uM Vem 1D_T-ERK” “1205_10uM Vem 1D_T-S6”

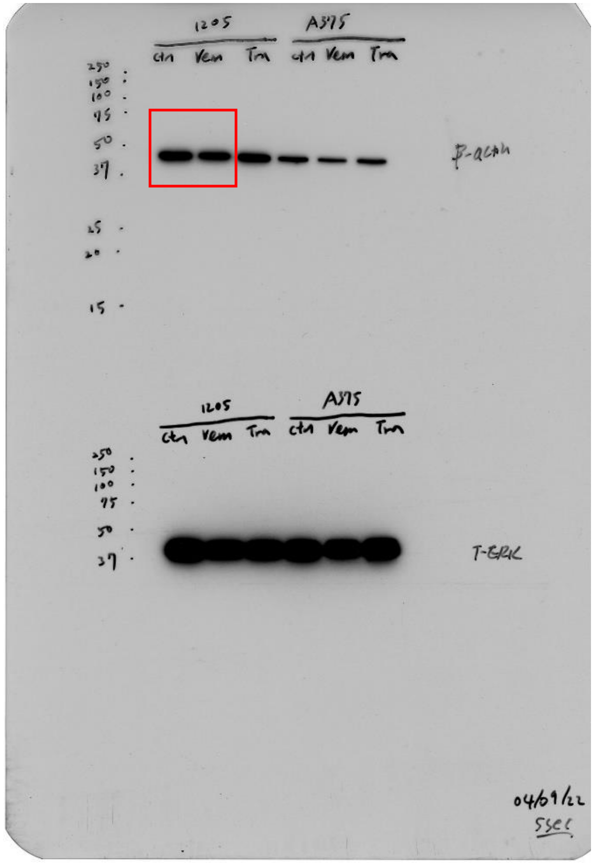

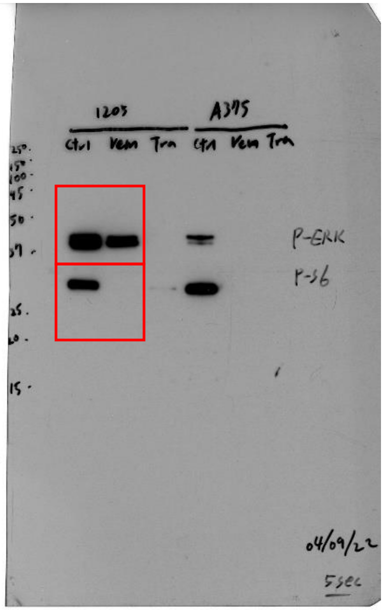

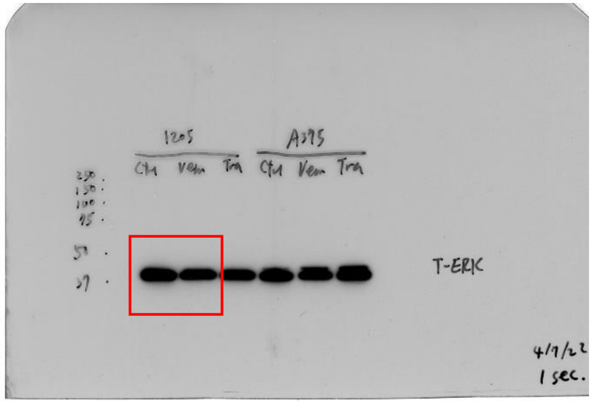

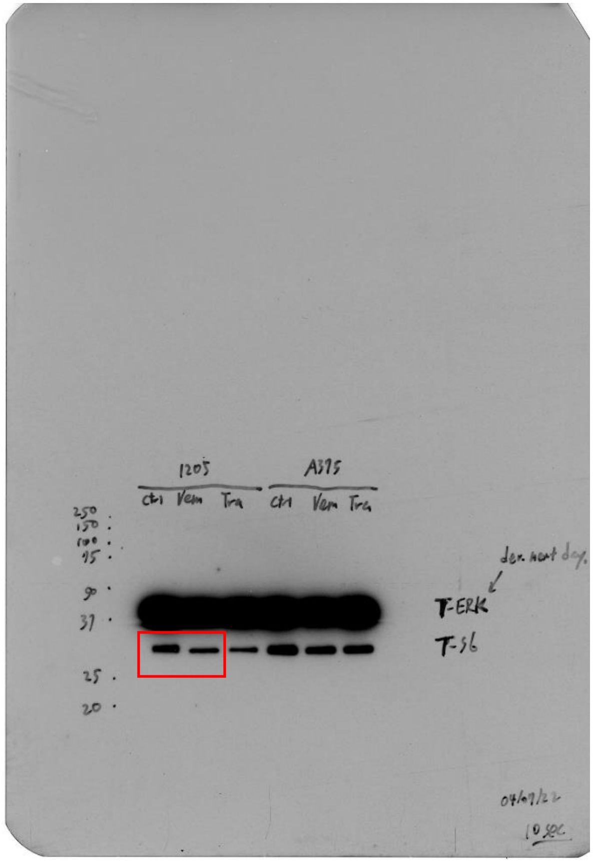

Supplemental Fig 4.

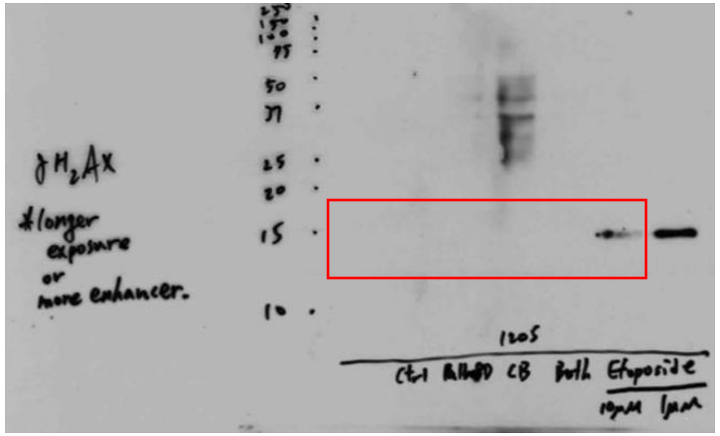

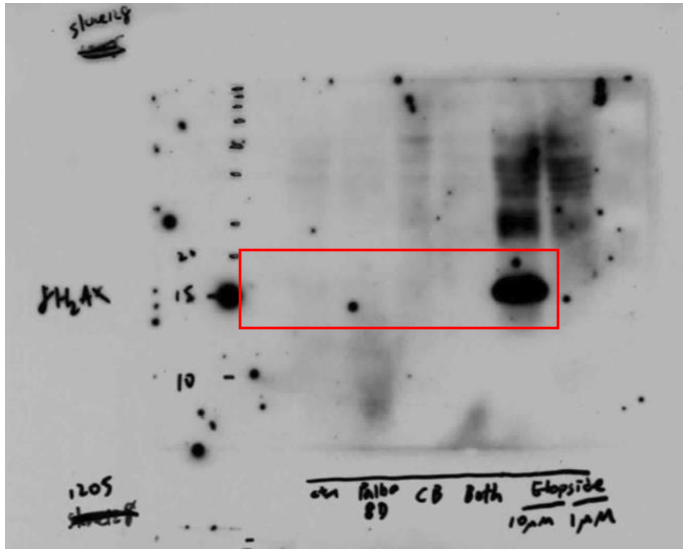

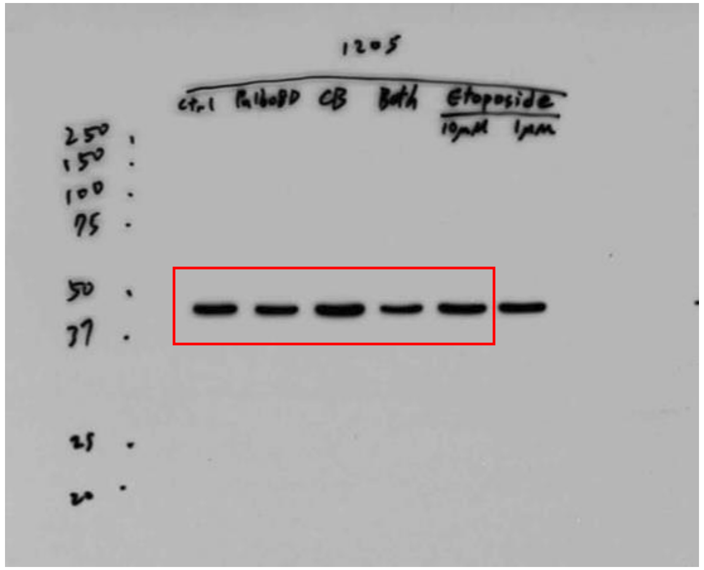

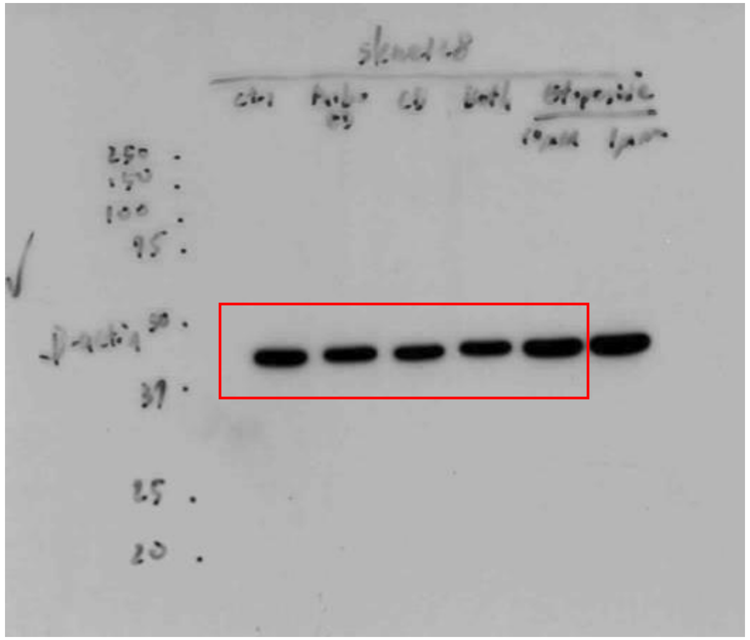

Supplemental Fig 5A.

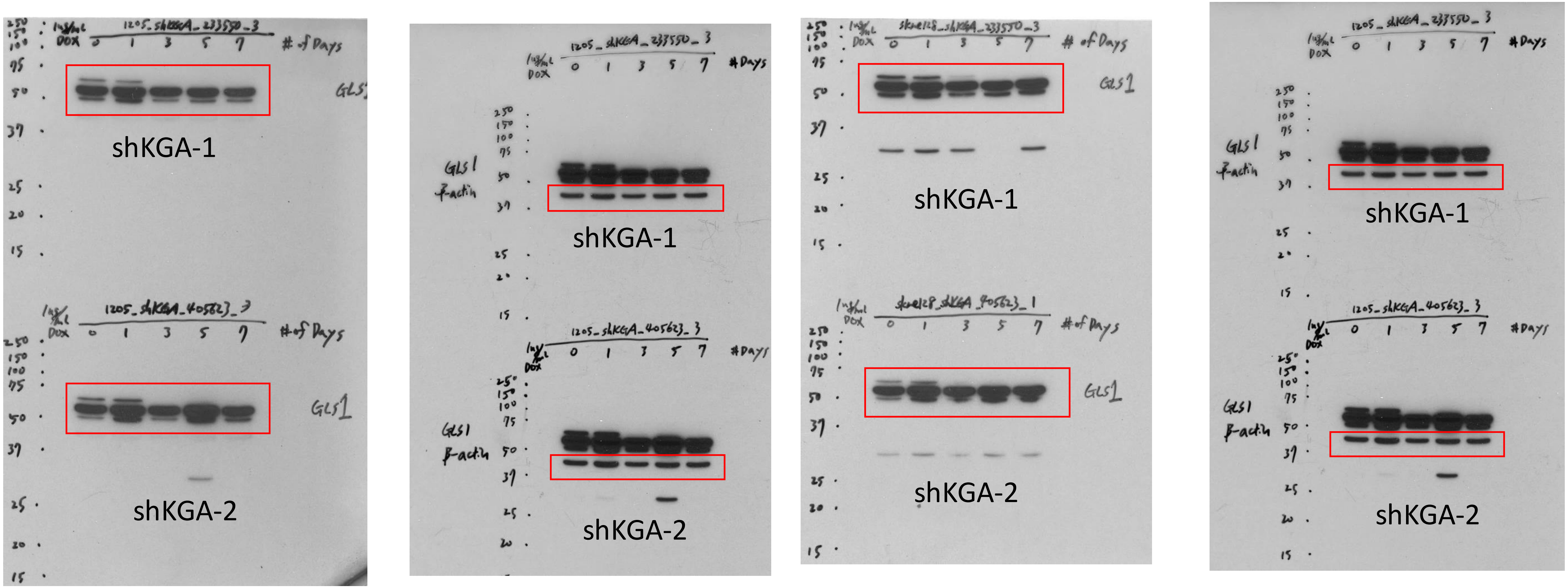

Supplemental Fig 5B.

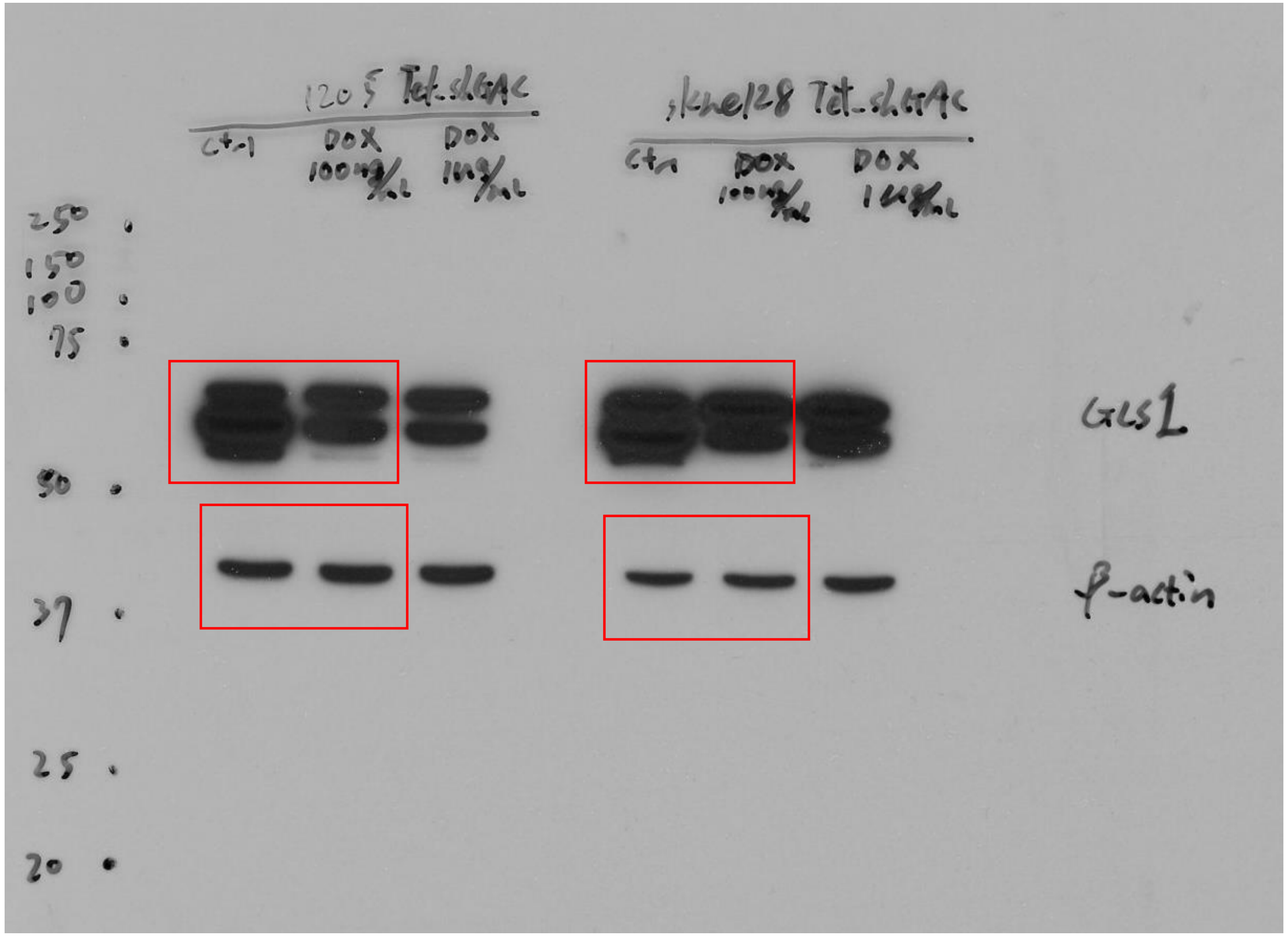

Supplemental Fig 6.

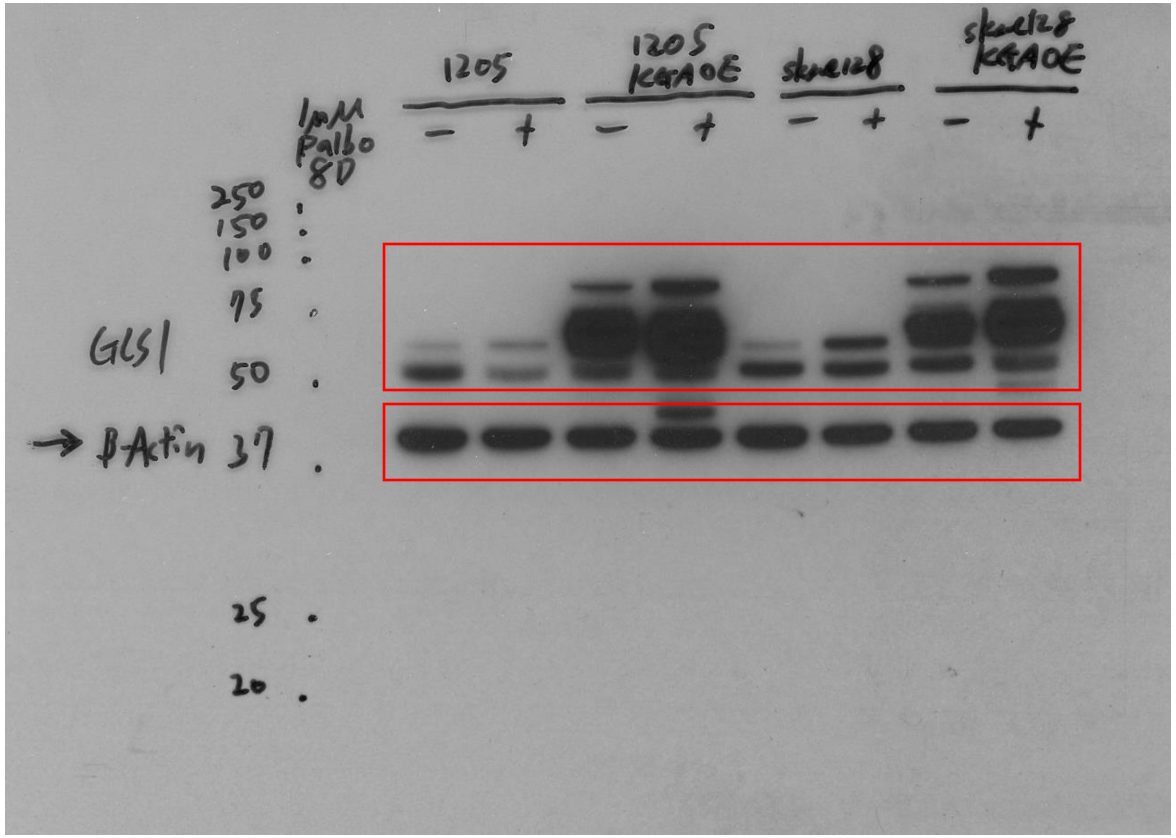

Supplement: Supplementary file 2 — Original western blots data [file 41419_2024_7284_MOESM2_ESM.pdf]
